# Management of Altered Passive Eruption With Gingivoplasty and Osteoplasty Prior to Esthetic Veneer Rehabilitation

**DOI:** 10.7759/cureus.114840

**Published:** 2026-08-20

**Authors:** Keily J Portillo Avila, Mercedes Dumont, Susana E Vivas M, Daniela Martinez, Mirelis Morciego Varona

**Affiliations:** 1 Periodontics, Dental Loft, San Pedro Sula, HND; 2 Dentistry, Family and Cosmetic Dentistry - Orlando, Orlando, USA; 3 Dentistry, Eastwood Dental, Orlando, USA; 4 Dentistry, Miramar Parkway Dental Care, Miramar, USA; 5 Dentistry, Dental Pearl, Miami, USA

**Keywords:** altered passive eruption, dental hypersensitivity, esthetic crown lengthening, esthetic dentistry, gingivoplasty, gummy smile, osteoplasty, porcelain veneers, smile design, veneer rehabilitation

## Abstract

Altered passive eruption is a common esthetic condition characterized by excessive gingival coverage of the anatomical crowns, resulting in short clinical crowns, excessive gingival display, and compromised smile esthetics. This case report describes the management of altered passive eruption in a 27-year-old male patient who presented with hypersensitivity affecting several maxillary anterior teeth, excessive gingival display, and dissatisfaction with the appearance of his smile. The patient reported that these conditions negatively affected his self-confidence in both social and professional settings. Clinical examination revealed short clinical crowns and unfavorable tooth proportions associated with excessive gingival tissue coverage. A treatment plan consisting of surgical smile design with gingivoplasty and osteoplasty was developed to establish appropriate gingival architecture and supracrestal tissue attachment prior to restorative treatment. Following flap elevation and osseous recontouring, favorable healing, improved gingival symmetry, and increased clinical crown length were achieved. After soft tissue maturation, esthetic veneer rehabilitation was completed to optimize tooth proportions and smile harmony. The combination of periodontal surgery and restorative treatment resulted in improved gingival contours and smile esthetics, while the patient reported resolution of the previously reported dental hypersensitivity and satisfaction with the treatment outcome. This case highlights the importance of a multidisciplinary approach in the management of altered passive eruption and illustrates how coordinated periodontal and restorative treatment can contribute to favorable short-term esthetic and clinical outcomes.

## Introduction

Altered passive eruption (APE) is a developmental condition characterized by excessive gingival coverage of the anatomical crowns, resulting in short clinical crowns and an altered relationship between the teeth and surrounding gingival tissues [1-5]. In APE, the gingival margin remains positioned more coronally than expected after tooth eruption, leaving part of the anatomical crown covered by gingival tissue. This condition may contribute to excessive gingival display, commonly referred to as a gummy smile, and can negatively affect smile esthetics and patient self-confidence [6-10]. In some cases, excessive gingival coverage may also be associated with increased plaque retention, difficulties in oral hygiene maintenance, and dental hypersensitivity [11,12].

Esthetic crown lengthening is a periodontal procedure used to correct excessive gingival display and improve clinical crown proportions [13-15]. Gingivoplasty involves recontouring the gingival tissues to establish a more favorable gingival margin and tooth contour, whereas osteoplasty involves recontouring the underlying alveolar bone when the osseous anatomy would otherwise interfere with establishment of adequate supracrestal tissue attachment. When indicated, combining these procedures can improve gingival symmetry and tooth proportions while creating a stable periodontal foundation for subsequent restorative treatment [16,17]. Careful evaluation of the gingival margin, cementoenamel junction (CEJ), and alveolar crest is therefore important when determining the appropriate treatment approach and achieving predictable esthetic and periodontal outcomes [18,19]. Although the management of APE is well established, selecting the appropriate combination and sequence of periodontal and restorative procedures remains clinically important because treatment must be individualized according to the periodontal anatomy and planned restorative outcome.

This report describes the management of a young adult patient presenting with dental hypersensitivity, excessive gingival display, and short clinical crowns associated with APE. An interdisciplinary treatment approach involving gingivoplasty and osteoplasty followed by tooth whitening and porcelain veneer rehabilitation was used to improve gingival architecture, tooth proportions, and overall smile esthetics [20].

## Case presentation

A 27-year-old male patient, classified as American Society of Anesthesiologists Physical Status I, with no known drug allergies, presented with sensitivity affecting several maxillary anterior teeth when exposed to cold and hot stimuli, as well as concerns regarding the appearance of his smile. A definitive isolated cause of the hypersensitivity could not be established clinically; therefore, it was documented as a concurrent presenting symptom rather than directly attributed to APE. Clinical examination revealed excessive gingival display and short clinical crowns, resulting in unfavorable tooth proportions and compromised smile esthetics. The patient reported that these conditions negatively affected his confidence in both social and professional settings. His primary goals were to reduce dental sensitivity, improve gingival symmetry and tooth proportions, and achieve a more harmonious and attractive smile through comprehensive esthetic rehabilitation.

**Figure 1:**
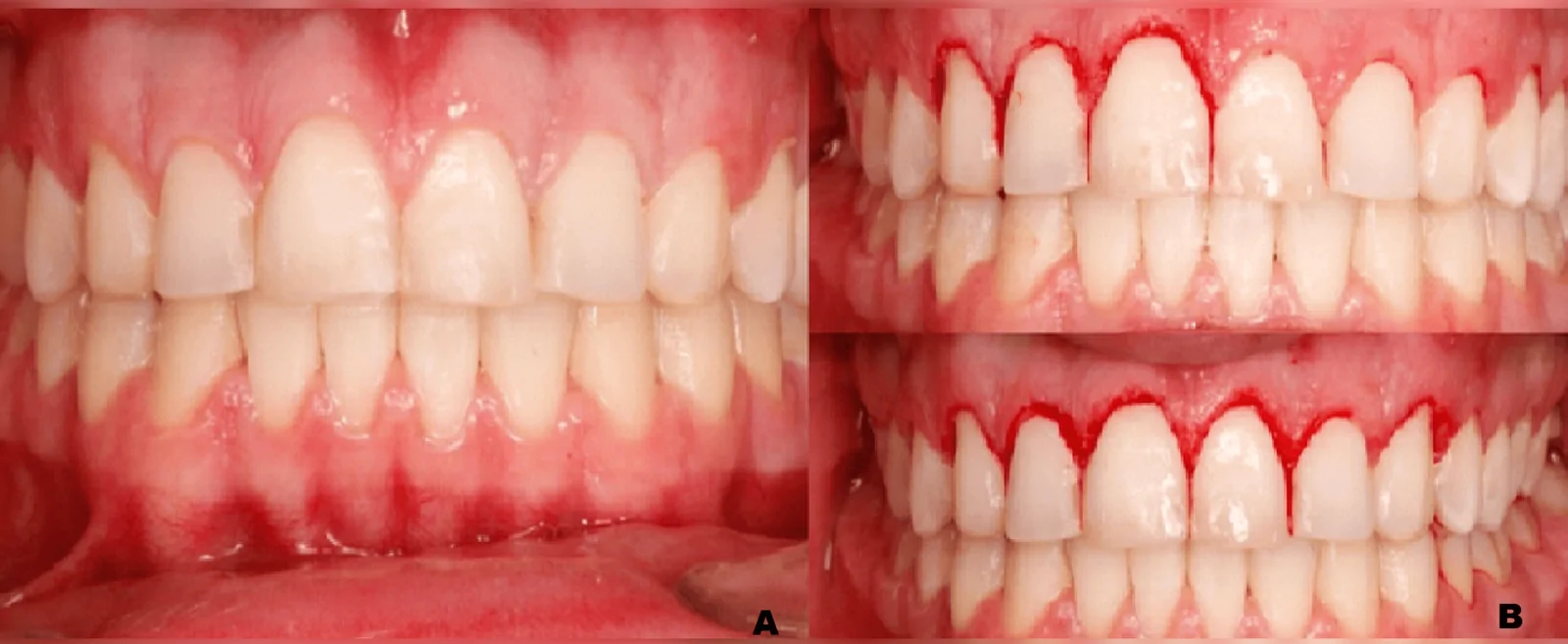
Preoperative clinical presentation and surgical crown-lengthening design in a patient with altered passive eruption. A: Preoperative frontal intraoral photograph demonstrating excessive gingival display and short clinical crowns associated with altered passive eruption. B: Intraoperative photographs showing the proposed gingival recontouring design marked prior to gingivoplasty and osteoplasty to establish appropriate gingival architecture and tooth proportions.

Clinical evaluation demonstrated excessive gingival coverage of the maxillary anterior teeth consistent with APE [1-5]. The gingival margins were positioned coronally relative to the CEJ, resulting in reduced clinical crown height and excessive gingival display during smiling. The periodontal tissues appeared healthy, with no clinical signs of active periodontal disease. Periodontal probing depths were within normal limits, and no clinical attachment loss, bleeding on probing, or pathologic tooth mobility was detected. Oral hygiene was considered satisfactory. The presentation represented excessive gingival coverage associated with APE rather than pathologic gingival overgrowth; therefore, a gingival overgrowth severity classification was not applicable. Based on the clinical findings and the patient's esthetic concerns, a treatment plan consisting of surgical smile design with gingivoplasty and osteoplasty, followed by esthetic rehabilitation with porcelain (ceramic) veneers, was developed. Periodontal surgery was planned to correct the excessive gingival coverage and establish stable gingival architecture, while subsequent veneer rehabilitation was intended to optimize the final tooth proportions and overall smile esthetics after periodontal healing (Figure 1).

Local anesthesia was achieved using 2% lidocaine with 1:100,000 epinephrine. Following administration of local anesthesia, gingivoplasty was performed in the maxillary anterior region to remove excessive gingival tissue and establish a more harmonious gingival contour. Bone sounding and clinical evaluation were used to assess the relationship among the gingival margin, CEJ, and alveolar crest and to evaluate the available space for supracrestal tissue attachment, historically referred to as biologic width [6-8]. Although tooth-specific numerical bone sounding measurements were not preserved in the available clinical record, the clinical findings indicated that soft tissue recontouring alone would not provide adequate space for supracrestal tissue attachment. A full-thickness flap was therefore elevated to permit direct visualization and evaluation of the underlying alveolar crest.

**Figure 2:**
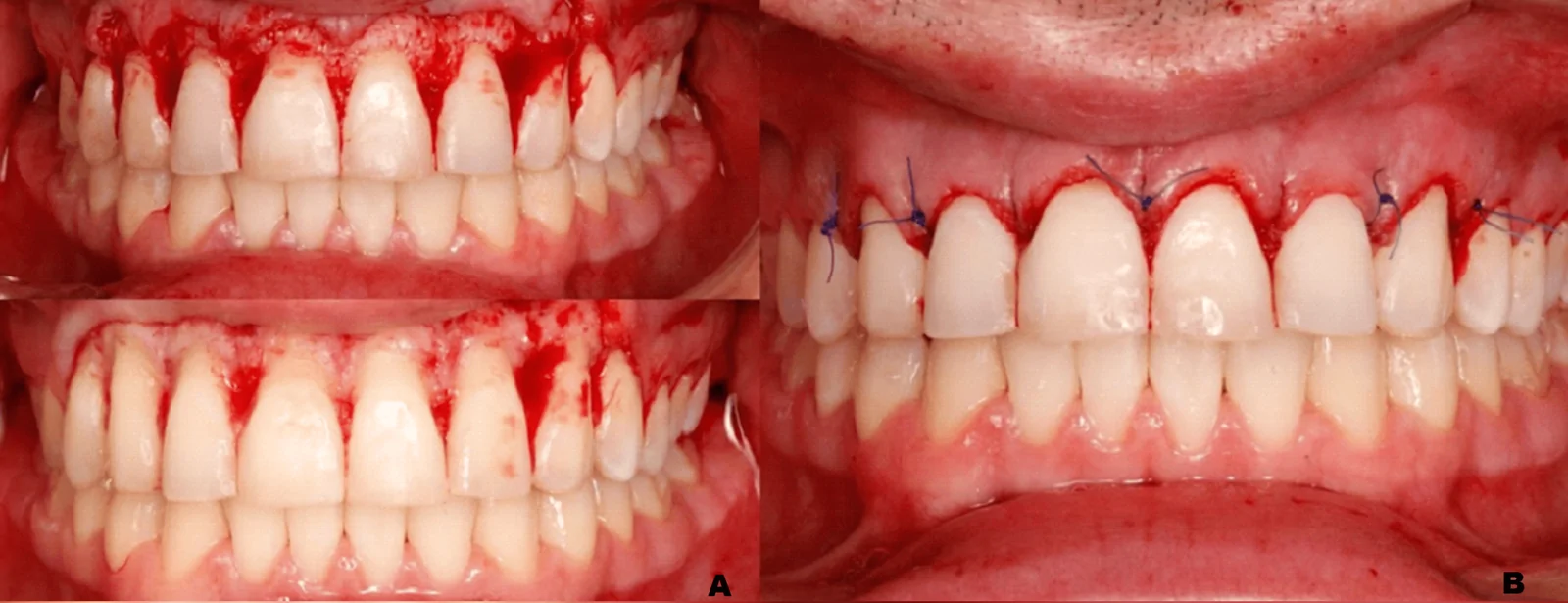
Surgical crown lengthening procedure and flap closure. A: Intraoperative photographs following gingivoplasty and full-thickness flap elevation, demonstrating exposure of the underlying alveolar crest and osseous recontouring performed to establish adequate supracrestal tissue attachment and appropriate crown length. B: Immediate postoperative clinical photograph showing flap repositioning and suturing after gingivoplasty and osteoplasty, with improved gingival architecture and increased clinical crown length. Flap closure was achieved using 3-0 resorbable catgut sutures.

Osteoplasty was subsequently performed to recontour the marginal bone and establish an appropriate relationship among the planned gingival margin, CEJ, and alveolar crest [9-15]. The amount of osseous recontouring was determined clinically according to the position of the CEJ, alveolar crest, and planned gingival margin, while preserving adequate space for supracrestal tissue attachment. Bone reduction was limited to the amount necessary to establish a favorable periodontal relationship and gingival architecture rather than being performed according to a predetermined uniform amount. The combination of gingivoplasty and osteoplasty was selected to improve clinical crown exposure and gingival symmetry while providing a stable periodontal foundation and reducing the risk of postoperative gingival rebound before definitive restorative treatment (Figure 2).

After completion of the osseous recontouring, the surgical site was irrigated, and the flap was repositioned and secured with interrupted 3-0 resorbable catgut sutures. Immediate postoperative evaluation demonstrated improved gingival architecture and increased clinical crown exposure (Figure 2).

Postoperative instructions were provided, and the patient was monitored during the healing phase. Healing progressed uneventfully, with progressive maturation of the periodontal tissues and improvement in gingival symmetry. An eight-week healing interval was allowed to permit adequate soft tissue healing, maturation, and stabilization of the gingival margins before definitive restorative treatment. At eight weeks, clinical evaluation demonstrated stable gingival margins and favorable tissue maturation, permitting progression to the restorative phase.

**Figure 3:**
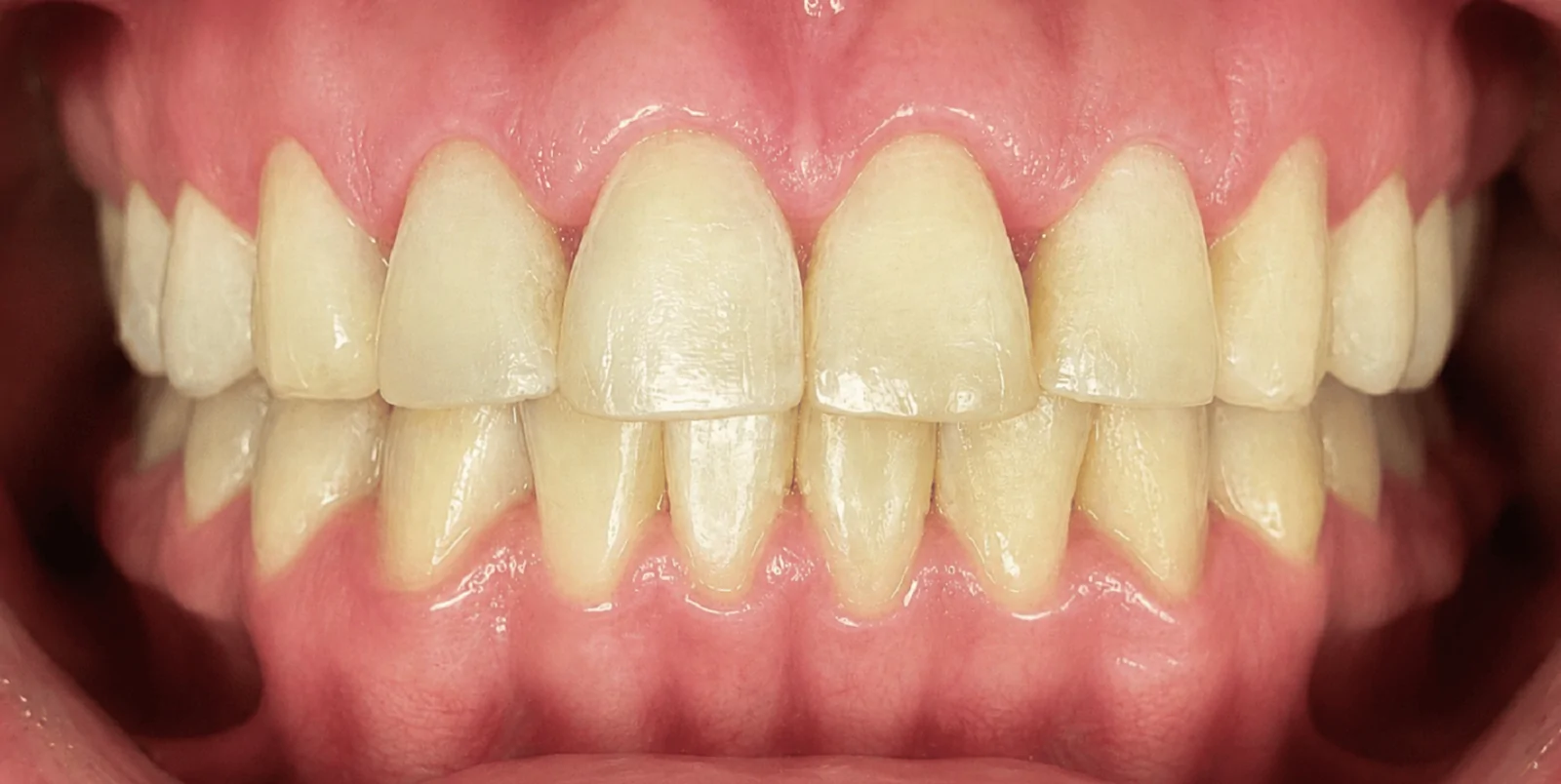
Healed gingival architecture following surgical crown lengthening and preparation for esthetic rehabilitation. Postoperative frontal intraoral photograph obtained after complete soft tissue healing following gingivoplasty and osteoplasty. The image demonstrates increased clinical crown length, improved gingival symmetry, and favorable tooth proportions achieved through surgical smile design. A minimally invasive facial reduction was subsequently performed on the maxillary anterior teeth in preparation for digital scanning and fabrication of definitive porcelain veneers.

Tooth whitening was performed before definitive restorative rehabilitation. Following whitening and stabilization of the periodontal tissues, minimally invasive facial preparation of the maxillary anterior teeth was performed, followed by digital scanning for fabrication of the definitive restorations (Figure 3).

**Figure 4:**
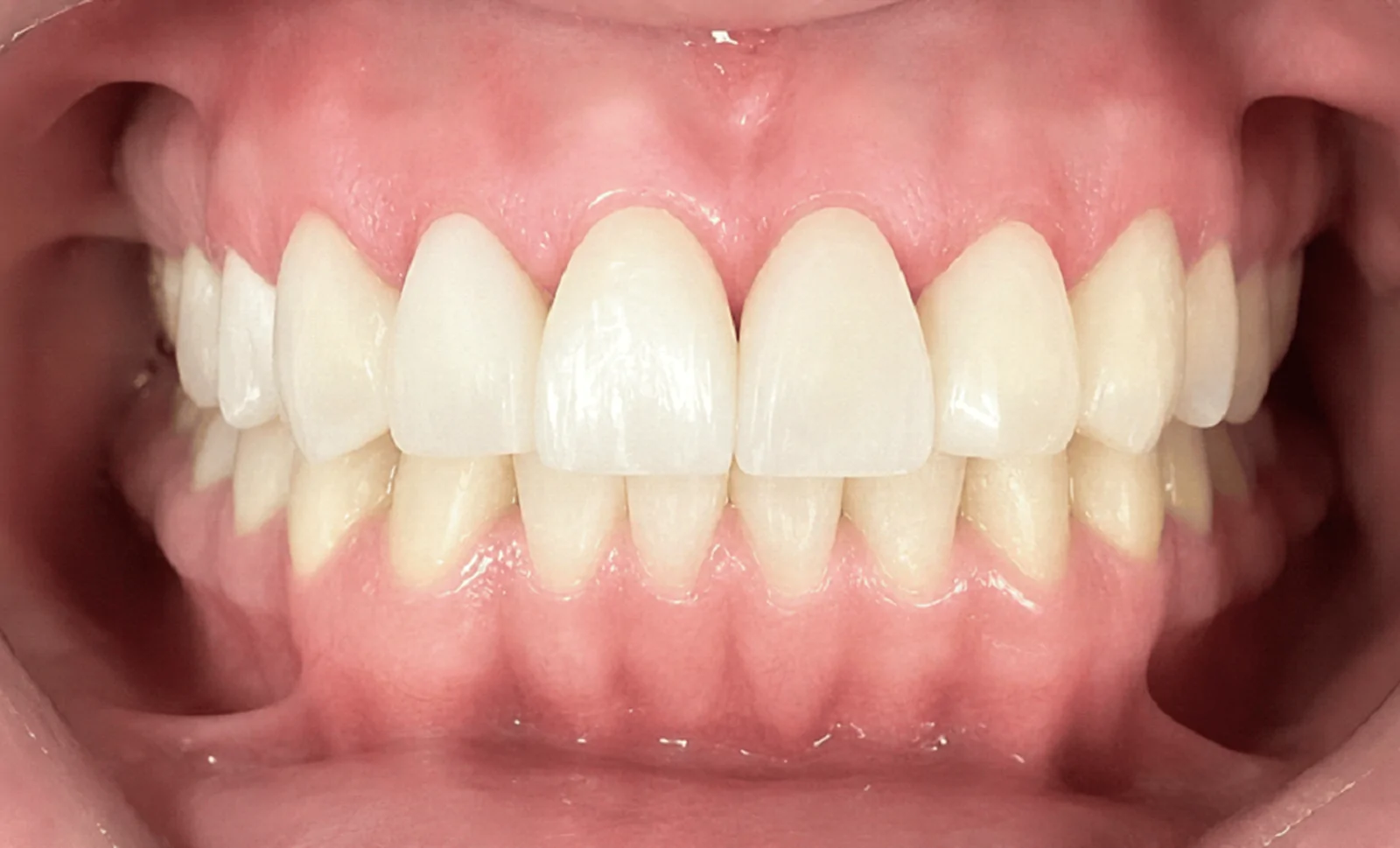
Three-week postoperative outcome following surgical crown lengthening and porcelain veneer rehabilitation. Clinical photograph obtained three weeks after placement of definitive porcelain veneers following gingivoplasty and osteoplasty. Improved gingival architecture, increased clinical crown length, enhanced tooth proportions, and harmonious integration of the periodontal and restorative components contributed to an esthetic smile outcome.

Porcelain (ceramic) veneers were subsequently fabricated and delivered. Detailed product-specific information regarding the whitening agent and the specific ceramic subtype was not available in the clinical record; therefore, these materials were not retrospectively specified. The completed periodontal and restorative treatment resulted in improved tooth proportions, harmonious gingival contours, and enhanced smile esthetics (Figure 4).

At the three-week follow-up visit after veneer placement, treatment success was evaluated clinically based on favorable periodontal healing, stable gingival margins, improved gingival symmetry and clinical crown exposure, satisfactory integration of the definitive porcelain (ceramic) veneers, absence of reported postoperative complications, and patient-reported outcomes. The gingival tissues demonstrated favorable healing and stable margins, and the definitive restorations showed satisfactory integration with the surrounding periodontal tissues. The patient reported satisfaction with the esthetic outcome and resolution of the previously reported dental hypersensitivity and subjectively described an improvement in self-confidence.

Written informed consent was obtained from the patient for publication of the clinical information and accompanying images. Formal ethical approval was not required for this single-patient case report according to the applicable institutional requirements.

## Discussion

APE is a common developmental condition in which the gingival margin fails to migrate apically to its normal position after tooth eruption, resulting in excessive gingival coverage of the anatomical crowns. Clinically, affected patients often present with short clinical crowns, excessive gingival display, and compromised smile esthetics [1-5]. In addition to esthetic concerns, some patients may report dental hypersensitivity and difficulties maintaining adequate oral hygiene; however, hypersensitivity should not be directly attributed to APE without identification of an underlying cause [6-8].

Successful management of APE requires careful evaluation of both the soft tissue and underlying osseous anatomy [9,10]. While gingivoplasty alone may improve gingival contours in selected cases, failure to address the relationship between the alveolar crest and the CEJ may result in inadequate supracrestal tissue attachment and an increased risk of gingival rebound [11,12]. Consequently, osteoplasty is frequently indicated when the osseous crest is positioned too coronally, allowing for the establishment of appropriate supracrestal tissue attachment and long-term periodontal stability [13-15].

In the present case, surgical smile design was selected primarily to address the patient's excessive gingival display, short clinical crowns, and esthetic concerns, while dental hypersensitivity was documented as a concurrent presenting symptom. Clinical evaluation revealed a coronally positioned alveolar crest, making osseous recontouring necessary to establish adequate supracrestal tissue attachment and long-term periodontal stability. The combination of gingivoplasty and osteoplasty resulted in increased clinical crown length, improved gingival symmetry, and more favorable tooth proportions. Establishing a stable periodontal foundation before restorative treatment was essential to achieving predictable esthetic results and minimizing the risk of future soft-tissue alterations. The favorable healing observed in this case is consistent with previous reports demonstrating that crown lengthening combined with osseous recontouring can provide stable periodontal outcomes when adequate supracrestal tissue attachment is established [16,17].

An interdisciplinary approach involving periodontal surgery, tooth whitening, and porcelain veneer rehabilitation was used to optimize the final outcome. Correcting the gingival architecture before veneer preparation allowed the definitive restorations to be designed according to ideal tooth proportions and smile characteristics. This sequence contributed to a more harmonious integration of the teeth, gingiva, and overall smile and facilitated a predictable esthetic outcome [18,19].

The treatment approach was selected according to the periodontal anatomy and the planned esthetic outcome. Gingival recontouring alone would not have addressed the coronally positioned alveolar crest identified clinically; therefore, osseous recontouring was incorporated to establish adequate space for supracrestal tissue attachment and reduce the potential for gingival rebound. Following periodontal stabilization, porcelain veneer rehabilitation was used to further optimize tooth proportions and overall smile esthetics. Although other approaches may be applicable to selected patients with excessive gingival display, the clinical findings in this case supported periodontal crown lengthening because the excessive gingival display was associated with excessive gingival coverage and the relationship between the gingival margin and underlying alveolar crest. The available clinical record did not document formal consideration of orthodontic treatment, composite bonding, botulinum toxin injection, or lip repositioning; therefore, these alternatives cannot be retrospectively presented as treatments that were specifically evaluated and rejected in this patient.

Dental hypersensitivity was a concurrent presenting symptom in this case; however, a definitive isolated cause could not be established clinically, and the symptom was therefore not directly attributed to APE. The patient reported resolution of hypersensitivity after completion of the periodontal and restorative treatment, but the relative contributions of crown lengthening, tooth whitening, and veneer rehabilitation to this improvement cannot be determined from this single case. Similarly, no conclusion can be drawn regarding whether tooth whitening produced transient sensitivity because this was not specifically documented. The available clinical record also did not document the use of specific desensitizing agents. Accordingly, the reported improvement in hypersensitivity should be interpreted as a patient-reported outcome following completion of the overall treatment rather than as evidence of a direct therapeutic effect of crown lengthening on hypersensitivity.

Although the individual periodontal and restorative procedures used in this case are well established, the clinical value of this report lies in the individualized, interdisciplinary treatment sequence used to manage APE in a patient presenting with esthetic concerns and concurrent dental hypersensitivity. Rather than relying on gingival recontouring alone, treatment incorporated clinical assessment of the gingival margin, CEJ, and alveolar crest to determine the need for combined gingivoplasty and osteoplasty, followed by an adequate healing period before tooth whitening and porcelain veneer rehabilitation. This coordinated sequence allowed stabilization of the periodontal tissues before definitive restorative treatment and resulted in improved gingival architecture, tooth proportions, and smile esthetics, together with patient satisfaction. The report therefore illustrates the importance of integrating periodontal and restorative planning according to patient-specific clinical findings rather than presenting a novel surgical technique.

This case report has limitations related to the available retrospective clinical documentation. Tooth-specific numerical periodontal and bone-sounding measurements, standardized preoperative and postoperative extraoral photographs, and radiographic images specifically documenting the alveolar crest before and after osseous recontouring were not available. Consequently, the diagnosis and treatment rationale are presented based on the documented clinical examination, bone sounding, available clinical photographs, and direct intraoperative assessment of the alveolar crest. These limitations should be considered when interpreting the reproducibility and outcomes of the treatment, and future reports would benefit from standardized periodontal measurements and comprehensive photographic and radiographic documentation.

The successful outcome observed in this case highlights the importance of accurate diagnosis and comprehensive treatment planning when managing APE. Addressing both the soft tissue and osseous components of the condition provided a stable foundation for subsequent esthetic rehabilitation. The patient expressed satisfaction with the final esthetic outcome and subjectively reported improvement in self-confidence; however, self-confidence was not assessed using a validated questionnaire and should therefore not be interpreted as an objectively measured psychosocial outcome [20].

## Conclusions

This case demonstrates a favorable short-term clinical outcome following multidisciplinary management of APE involving gingivoplasty, osteoplasty, tooth whitening, and porcelain veneer rehabilitation. Correction of the excessive gingival display and short clinical crowns resulted in improved gingival symmetry, increased clinical crown exposure, and more favorable tooth proportions. Osseous recontouring was performed to establish an appropriate relationship between the alveolar crest and the planned gingival margin before the subsequent restorative phase.

The combination of periodontal surgery and esthetic rehabilitation resulted in improved smile esthetics and satisfaction with the treatment outcome, and the patient reported resolution of the previously reported dental hypersensitivity. Although the patient subjectively reported an improvement in self-confidence following treatment, this outcome was not assessed using a validated questionnaire and therefore should not be interpreted as an objectively measured psychosocial outcome. Given the single-patient nature of this report and the relatively short follow-up period, long-term stability and generalizability cannot be established. This case highlights the importance of comprehensive diagnosis and coordinated periodontal restorative treatment planning in the management of APE.
